# *catena*-Poly[[(8-amino­quinoline-κ^2^ *N*,*N*′)cadmium]-di-μ-thio­cyanato-κ^2^ *N*:*S*;κ^2^ *S*:*N*-[(8-amino­quinoline-κ^2^ *N*,*N*′)cadmium]-di-μ-chlorido]

**DOI:** 10.1107/S1600536811051373

**Published:** 2011-12-03

**Authors:** Heng Xu, Chang Guo

**Affiliations:** a Anhui Key Laboratory of Functional Coordination Compounds, School of Chemistry and Chemical Engineering, Anqing Normal University, Anqing 246011, People’s Republic of China

## Abstract

In the title compound, [CdCl(NCS)(C_9_H_8_N_2_)]_*n*_, the Cd^II^ atom is in a distorted octa­hedral coordination environment defined by two chloride anions, two N atoms from an 8-amino­quinoline ligand, one N atom from one thio­cyanate anion and one S atom from a symmetry-related thio­cyanate anion. Two Cd^II^ atoms are bridged by two chloride anions, forming an inversion-related Cd_2_Cl_2_ unit; these units are further linked through thio­cyanate anions, leading to a chain structure extending parallel to [010]. Weak π–π stacking inter­actions with centroid–centroid distances of 3.430 (4) Å and an inter­planar separation of 3.390 (3) Å between the pyridine and benzene rings link the chains into a two-dimensional network parallel to (10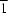). Weak inter­molecular C—H⋯Cl hydrogen-bonding inter­actions help to consolidate the crystal packing.

## Related literature

For background and applications of 8-amino­quinoline and its derivatives, see: Fritsch *et al.* (2006 ▶); Kim *et al.* (2004 ▶); Li *et al.* (2005 ▶); Macias *et al.* (2003 ▶); Bortoluzzi *et al.* (2006 ▶); Tekwami & Walker (2006 ▶).
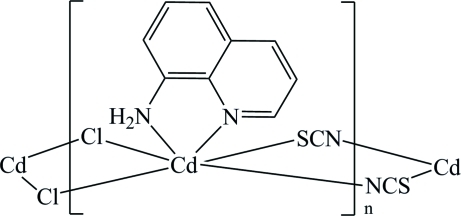


## Experimental

### 

#### Crystal data

[CdCl(NCS)(C_9_H_8_N_2_)]
*M* *_r_* = 350.10
Triclinic, 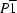

*a* = 7.4965 (6) Å
*b* = 8.6245 (7) Å
*c* = 10.5247 (12) Å
α = 106.649 (7)°
β = 98.047 (7)°
γ = 112.561 (5)°
*V* = 577.53 (9) Å^3^
*Z* = 2
Mo *K*α radiation
μ = 2.28 mm^−1^
*T* = 293 K
0.22 × 0.20 × 0.18 mm

#### Data collection

Bruker SMART CCD area-detector diffractometer
Absorption correction: multi-scan (*SADABS*; Bruker, 2000 ▶) *T*_min_ = 0.635, *T*_max_ = 0.685
5150 measured reflections
2104 independent reflections
1961 reflections with *I* > 2σ(*I*)
*R*_int_ = 0.021

#### Refinement

*R*[*F* ^2^ > 2σ(*F* ^2^)] = 0.021
*wR*(*F* ^2^) = 0.051
*S* = 1.04
2104 reflections
153 parameters
H atoms treated by a mixture of independent and constrained refinement
Δρ_max_ = 0.74 e Å^−3^
Δρ_min_ = −0.50 e Å^−3^

### 

Data collection: *SMART* (Bruker, 2000 ▶); cell refinement: *SAINT* (Bruker, 2000 ▶); data reduction: *SAINT*; program(s) used to solve structure: *SHELXS97* (Sheldrick, 2008 ▶); program(s) used to refine structure: *SHELXL97* (Sheldrick, 2008 ▶); molecular graphics: *SHELXTL* (Sheldrick, 2008 ▶); software used to prepare material for publication: *publCIF* (Westrip, 2010 ▶).

## Supplementary Material

Crystal structure: contains datablock(s) I, global. DOI: 10.1107/S1600536811051373/wm2567sup1.cif

Structure factors: contains datablock(s) I. DOI: 10.1107/S1600536811051373/wm2567Isup2.hkl

Additional supplementary materials:  crystallographic information; 3D view; checkCIF report

## Figures and Tables

**Table 1:** Selected bond lengths (Å)

Cd1—N3^i^	2.311 (2)
Cd1—N1	2.322 (2)
Cd1—N2	2.382 (3)
Cd1—Cl1	2.5495 (8)
Cd1—S1	2.6413 (8)
Cd1—Cl1^ii^	2.8088 (7)

Symmetry codes: (i) 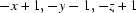; (ii) 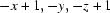.

**Table 2:** Hydrogen-bond geometry (Å, °)

*D*—H⋯*A*	*D*—H	H⋯*A*	*D*⋯*A*	*D*—H⋯*A*
C2—H2⋯Cl1^iii^	0.93	2.84	3.723 (4)	160

Symmetry code: (iii) 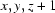.

**Fig. 1.**
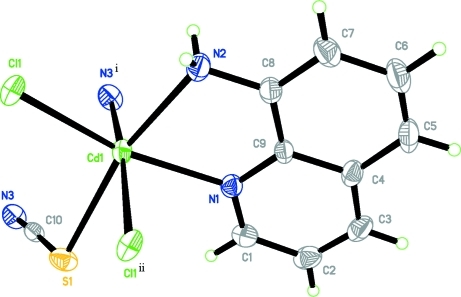
The molecular structure of the title compound, showing the atom-labelling scheme and displacement ellipsoids drawn at the 30% probability level. [Symmetry codes: (i) -x + 1, -y - 1, -z + 1; (ii) -x + 1, -y, -z + 1.]

**Fig. 2.**
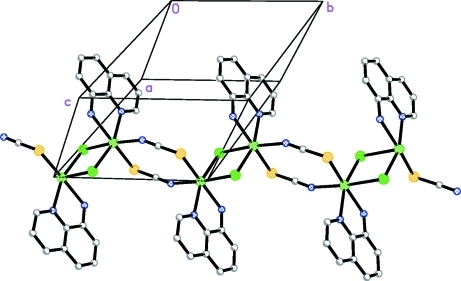
The crystal packing of the title compound, showing the chain structure extending parallel to [010].

**Fig. 3.**
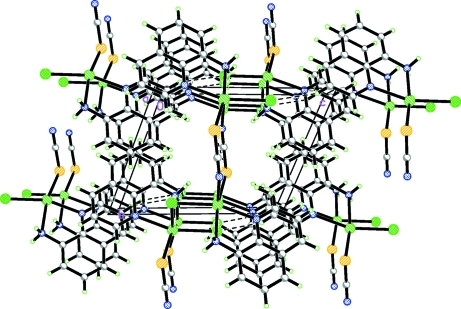
A view showing part of the three-dimensional supramolecular network linked by C–H···Cl hydrogen bonds and weak π—π stacking interactions. Hydrogen bonds are shown as dashed lines.

## supplementary crystallographic information

### Comment

8-aminoquinoline and its derivatives are systems that have been recently focused on, because of antiprotozal and other pharmaceutical properties (Tekwami & Walker, 2006). They are also strongly fluorescent and have been employed in the analytical study of heavy metals (Fritsch *et al.*, 2006; Macias *et al.*, 2003). They also have been used to prepare highly conducting co-polymers (Li *et al.*, 2005). Different functionalized molecules of 8- aminoquinoline have been recently reported (Kim *et al.*, 2004). However, the coordination chemistry of 8-aminoquinoline, as such, is scarce (Bortoluzzi *et al.*, 2006). We report here the crystal structure of the title compound, [CdCl(SCN)(C_9_H_8_N_2_)]_n_, (I).

As shown in Fig. 1, the asymmetric unit of (I) contains one Cd^II^ cation, one chloride anion and one thiocyanate anion. Each Cd^II^ cation is in a distorted octahedral coordination environment defined by two chloride anions, two nitrogen atoms from one 8-aminoquinoline ligand, one nitrogen atom from one thiocyanate anion and one sulfur atom from another thiocyanate anion. Two Cd^II^ atoms are connected by two chloride anions to form a dimer and these dimers are further bridged through two thiocyanate anions, leading to a chain structure extending parallel to [010] (Fig. 2). Moreover, weak π-π stacking interactions (centroid···centroid distances of 3.430 (4) Å and an interplanar separation of 3.390 (3) Å between pyridyl rings and benzene rings) link the chains into a two-dimensional supramolecular network in the (101) plane, which is further consolidated by intermolecular C—H···Cl hydrogen bonds to genrate a three-dimensional supramolecular structure (Fig. 3). It is interesting to note that the amino hydrogen atoms are not involved in any hydrogen bonding interactions.

### Experimental

8-aminoquinoline (1 mmol) and potassium thiocyanate (1 mmol) in 20 ml methanol were added to a clear solution of cadmium chloride (1 mmol) in 20 ml methanol. Stirring was continued for 1 h; the colour changed to light yellow. The volume of the solution was reduced to 10 ml, filtered and kept for crystallization after addition of 2 drops of 2-methoxy ethanol. Colorless block-like crystals were obtained by slow evaporation of the solvent. Yield: 59%.

### Refinement

All hydrogen atoms bonded to carbon were positioned geometrically and refined using a riding model, with C—H = 0.93 Å and with *U*_iso_(H) = 1.2*U*_eq_(C). The amino H atoms were found from difference maps and were refined with distance restraint of N—H = 0.86 Å and *U*_iso_(H) = 1.2*U*_eq_(N).

### Crystal data

**Table:**

[CdCl(NCS)(C_9_H_8_N_2_)]	*Z* = 2
*M**_r_* = 350.10	*F*(000) = 340
Triclinic, *P*1	*D*_x_ = 2.013 Mg m^−^^3^
Hall symbol: -P 1	Mo *K*α radiation, λ = 0.71073 Å
*a* = 7.4965 (6) Å	Cell parameters from 5150 reflections
*b* = 8.6245 (7) Å	θ = 2.1–25.5°
*c* = 10.5247 (12) Å	µ = 2.28 mm^−^^1^
α = 106.649 (7)°	*T* = 293 K
β = 98.047 (7)°	Block, colorless
γ = 112.561 (5)°	0.22 × 0.20 × 0.18 mm
*V* = 577.53 (9) Å^3^	

### Data collection

**Table:**

Bruker SMART CCD area-detector diffractometer	2104 independent reflections
Radiation source: sealed tube	1961 reflections with *I* > 2σ(*I*)
graphite	*R*_int_ = 0.021
phi and ω scans	θ_max_ = 25.5°, θ_min_ = 2.1°
Absorption correction: multi-scan (*SADABS*; Bruker, 2000)	*h* = −9→9
*T*_min_ = 0.635, *T*_max_ = 0.685	*k* = −10→10
5150 measured reflections	*l* = −12→12

### Refinement

**Table:**

Refinement on *F*^2^	Primary atom site location: structure-invariant direct methods
Least-squares matrix: full	Secondary atom site location: difference Fourier map
*R*[*F*^2^ > 2σ(*F*^2^)] = 0.021	Hydrogen site location: inferred from neighbouring sites
*wR*(*F*^2^) = 0.051	H atoms treated by a mixture of independent and constrained refinement
*S* = 1.04	*w* = 1/[σ^2^(*F*_o_^2^) + (0.0243*P*)^2^ + 0.2713*P*] where *P* = (*F*_o_^2^ + 2*F*_c_^2^)/3
2104 reflections	(Δ/σ)_max_ = 0.002
153 parameters	Δρ_max_ = 0.74 e Å^−^^3^
0 restraints	Δρ_min_ = −0.50 e Å^−^^3^

### Special details

**Table:**

Geometry. All e.s.d.'s (except the e.s.d. in the dihedral angle between two l.s. planes) are estimated using the full covariance matrix. The cell e.s.d.'s are taken into account individually in the estimation of e.s.d.'s in distances, angles and torsion angles; correlations between e.s.d.'s in cell parameters are only used when they are defined by crystal symmetry. An approximate (isotropic) treatment of cell e.s.d.'s is used for estimating e.s.d.'s involving l.s. planes.
Refinement. Refinement of *F*^2^ against ALL reflections. The weighted *R*-factor *wR* and goodness of fit *S* are based on *F*^2^, conventional *R*-factors *R* are based on *F*, with *F* set to zero for negative *F*^2^. The threshold expression of *F*^2^ > σ(*F*^2^) is used only for calculating *R*-factors(gt) *etc*. and is not relevant to the choice of reflections for refinement. *R*-factors based on *F*^2^ are statistically about twice as large as those based on *F*, and *R*- factors based on ALL data will be even larger.

### Fractional atomic coordinates and isotropic or equivalent isotropic displacement parameters (Å^2^)

**Table:**

	*x*	*y*	*z*	*U*_iso_*/*U*_eq_	
Cd1	0.62856 (3)	−0.10583 (2)	0.594940 (19)	0.03402 (8)	
Cl1	0.56554 (12)	−0.11665 (9)	0.34743 (7)	0.04336 (17)	
N1	0.7549 (3)	0.0017 (3)	0.8336 (2)	0.0356 (5)	
N2	0.9449 (5)	0.1523 (4)	0.6597 (3)	0.0515 (7)	
N3	0.2515 (4)	−0.6802 (3)	0.4552 (3)	0.0456 (6)	
S1	0.26442 (11)	−0.34111 (9)	0.57610 (9)	0.0464 (2)	
C1	0.6679 (4)	−0.0751 (4)	0.9132 (3)	0.0431 (7)	
H1	0.5546	−0.1865	0.8719	0.052*	
C2	0.7371 (5)	0.0024 (5)	1.0580 (3)	0.0518 (8)	
H2	0.6710	−0.0569	1.1110	0.062*	
C3	0.9004 (5)	0.1638 (5)	1.1190 (3)	0.0525 (8)	
H3	0.9471	0.2172	1.2149	0.063*	
C4	1.0010 (4)	0.2524 (4)	1.0383 (3)	0.0425 (7)	
C5	1.1737 (5)	0.4201 (4)	1.0939 (3)	0.0576 (9)	
H5	1.2259	0.4794	1.1893	0.069*	
C6	1.2648 (5)	0.4961 (4)	1.0102 (4)	0.0610 (9)	
H6	1.3781	0.6077	1.0487	0.073*	
C7	1.1906 (5)	0.4087 (4)	0.8661 (4)	0.0526 (8)	
H7	1.2563	0.4622	0.8101	0.063*	
C8	1.0226 (4)	0.2458 (4)	0.8069 (3)	0.0394 (6)	
C9	0.9235 (4)	0.1647 (3)	0.8922 (3)	0.0322 (6)	
C10	0.2613 (4)	−0.5392 (4)	0.5052 (3)	0.0344 (6)	
H8A	0.925 (7)	0.225 (6)	0.623 (5)	0.097 (16)*	
H8B	1.026 (8)	0.108 (7)	0.629 (5)	0.110 (19)*	

### Atomic displacement parameters (Å^2^)

**Table:**

	*U*^11^	*U*^22^	*U*^33^	*U*^12^	*U*^13^	*U*^23^
Cd1	0.03527 (12)	0.03000 (12)	0.02813 (13)	0.01083 (9)	0.00321 (9)	0.00685 (9)
Cl1	0.0617 (4)	0.0430 (4)	0.0277 (4)	0.0279 (3)	0.0119 (3)	0.0102 (3)
N1	0.0353 (12)	0.0390 (12)	0.0320 (12)	0.0179 (10)	0.0070 (10)	0.0116 (10)
N2	0.0512 (16)	0.0495 (16)	0.0336 (15)	0.0056 (13)	0.0091 (13)	0.0125 (13)
N3	0.0408 (13)	0.0354 (13)	0.0548 (16)	0.0181 (11)	0.0078 (12)	0.0098 (12)
S1	0.0342 (4)	0.0322 (3)	0.0633 (5)	0.0124 (3)	0.0163 (4)	0.0069 (3)
C1	0.0396 (15)	0.0500 (17)	0.0483 (18)	0.0219 (14)	0.0149 (14)	0.0263 (15)
C2	0.0520 (19)	0.081 (2)	0.0423 (19)	0.0389 (19)	0.0210 (16)	0.0334 (18)
C3	0.057 (2)	0.081 (2)	0.0314 (16)	0.047 (2)	0.0127 (15)	0.0168 (16)
C4	0.0407 (15)	0.0524 (17)	0.0318 (15)	0.0298 (14)	0.0017 (13)	0.0036 (13)
C5	0.0508 (19)	0.0547 (19)	0.0415 (19)	0.0243 (16)	−0.0083 (16)	−0.0088 (16)
C6	0.0453 (18)	0.0422 (17)	0.061 (2)	0.0074 (15)	−0.0031 (17)	−0.0022 (16)
C7	0.0433 (17)	0.0392 (16)	0.058 (2)	0.0078 (14)	0.0083 (16)	0.0125 (15)
C8	0.0376 (15)	0.0378 (14)	0.0349 (16)	0.0146 (12)	0.0050 (13)	0.0082 (12)
C9	0.0302 (13)	0.0328 (13)	0.0285 (14)	0.0161 (11)	0.0021 (11)	0.0043 (11)
C10	0.0237 (12)	0.0378 (15)	0.0366 (15)	0.0096 (11)	0.0064 (11)	0.0137 (12)

### Geometric parameters (Å, °)

**Table:**

Cd1—N3^i^	2.311 (2)	C1—C2	1.402 (4)
Cd1—N1	2.322 (2)	C1—H1	0.9300
Cd1—N2	2.382 (3)	C2—C3	1.345 (5)
Cd1—Cl1	2.5495 (8)	C2—H2	0.9300
Cd1—S1	2.6413 (8)	C3—C4	1.408 (5)
Cd1—Cl1^ii^	2.8088 (7)	C3—H3	0.9300
Cl1—Cd1^ii^	2.8088 (7)	C4—C5	1.406 (5)
N1—C1	1.306 (4)	C4—C9	1.421 (4)
N1—C9	1.370 (3)	C5—C6	1.352 (5)
N2—C8	1.436 (4)	C5—H5	0.9300
N2—H8A	0.87 (5)	C6—C7	1.402 (5)
N2—H8B	0.88 (5)	C6—H6	0.9300
N3—C10	1.147 (3)	C7—C8	1.367 (4)
N3—Cd1^i^	2.311 (2)	C7—H7	0.9300
S1—C10	1.646 (3)	C8—C9	1.413 (4)
			
N3^i^—Cd1—N1	96.56 (8)	N1—C1—H1	118.4
N3^i^—Cd1—N2	96.66 (11)	C2—C1—H1	118.4
N1—Cd1—N2	72.51 (9)	C3—C2—C1	119.0 (3)
N3^i^—Cd1—Cl1	92.72 (7)	C3—C2—H2	120.5
N1—Cd1—Cl1	161.21 (6)	C1—C2—H2	120.5
N2—Cd1—Cl1	90.24 (7)	C2—C3—C4	120.3 (3)
N3^i^—Cd1—S1	94.00 (6)	C2—C3—H3	119.9
N1—Cd1—S1	97.09 (6)	C4—C3—H3	119.9
N2—Cd1—S1	165.86 (8)	C5—C4—C3	123.8 (3)
Cl1—Cd1—S1	98.53 (3)	C5—C4—C9	118.6 (3)
N3^i^—Cd1—Cl1^ii^	172.43 (6)	C3—C4—C9	117.5 (3)
N1—Cd1—Cl1^ii^	84.41 (6)	C6—C5—C4	120.7 (3)
N2—Cd1—Cl1^ii^	90.80 (10)	C6—C5—H5	119.6
Cl1—Cd1—Cl1^ii^	88.55 (2)	C4—C5—H5	119.6
S1—Cd1—Cl1^ii^	78.43 (2)	C5—C6—C7	120.8 (3)
Cd1—Cl1—Cd1^ii^	91.45 (2)	C5—C6—H6	119.6
C1—N1—C9	119.4 (2)	C7—C6—H6	119.6
C1—N1—Cd1	124.5 (2)	C8—C7—C6	120.8 (3)
C9—N1—Cd1	115.88 (17)	C8—C7—H7	119.6
C8—N2—Cd1	112.08 (19)	C6—C7—H7	119.6
C8—N2—H8A	109 (3)	C7—C8—C9	119.5 (3)
Cd1—N2—H8A	107 (3)	C7—C8—N2	121.9 (3)
C8—N2—H8B	109 (3)	C9—C8—N2	118.6 (2)
Cd1—N2—H8B	105 (3)	N1—C9—C8	119.8 (2)
H8A—N2—H8B	116 (4)	N1—C9—C4	120.6 (3)
C10—N3—Cd1^i^	156.0 (2)	C8—C9—C4	119.6 (3)
C10—S1—Cd1	104.03 (9)	N3—C10—S1	177.4 (2)
N1—C1—C2	123.1 (3)		
			
C9—N1—C1—C2	0.9 (4)	Cd1—N2—C8—C9	8.6 (4)
Cd1—N1—C1—C2	−173.5 (2)	C1—N1—C9—C8	177.8 (2)
N1—C1—C2—C3	0.2 (5)	Cd1—N1—C9—C8	−7.3 (3)
C1—C2—C3—C4	−0.6 (5)	C1—N1—C9—C4	−1.6 (4)
C2—C3—C4—C5	−179.3 (3)	Cd1—N1—C9—C4	173.23 (18)
C2—C3—C4—C9	−0.1 (4)	C7—C8—C9—N1	179.8 (3)
C3—C4—C5—C6	178.9 (3)	N2—C8—C9—N1	−1.2 (4)
C9—C4—C5—C6	−0.2 (4)	C7—C8—C9—C4	−0.7 (4)
C4—C5—C6—C7	−0.7 (5)	N2—C8—C9—C4	178.3 (3)
C5—C6—C7—C8	0.9 (5)	C5—C4—C9—N1	−179.6 (3)
C6—C7—C8—C9	−0.2 (5)	C3—C4—C9—N1	1.2 (4)
C6—C7—C8—N2	−179.2 (3)	C5—C4—C9—C8	1.0 (4)
Cd1—N2—C8—C7	−172.4 (2)	C3—C4—C9—C8	−178.2 (2)

Symmetry codes: (i) −*x*+1, −*y*−1, −*z*+1; (ii) −*x*+1, −*y*, −*z*+1.

### Hydrogen-bond geometry (Å, °)

**Table:**

*D*—H···*A*	*D*—H	H···*A*	*D*···*A*	*D*—H···*A*
C2—H2···Cl1^iii^	0.93	2.84	3.723 (4)	160

Symmetry codes: (iii) *x*, *y*, *z*+1.
